# Determinants of Exclusive Breastfeeding and Introduction of Complementary foods in Rural Egyptian Communities

**DOI:** 10.5539/gjhs.v6n4p236

**Published:** 2014-04-20

**Authors:** Arwa Mohamed Hosney El Shafei, John Rene Labib

**Affiliations:** 1 Lecturer of Public Health and Community Medicine, Kasr Al Aini Faculty of Medicine, Cairo University, Cairo, Egypt; 2 Lecturer of Pediatrics, Kasr Al Aini Faculty of Medicine, Cairo University, Cairo, Egypt

**Keywords:** breastfeeding, exclusive breastfeeding, complementary feeding, rural Egypt

## Abstract

**Background:**

Determinants of breastfeeding (BF) exclusivity at a rural Egyptian setting are largely unknown. This cross-sectional study aimed to assess BF indicators, specifically exclusivity and the timely complementary feeding while assessing potential determinants that affect exclusivity of BF among a sample of mothers inhabiting Egyptian rural communities.

**Methods:**

A community based cross-sectional study was carried out over a period of four months with inclusion of 1000 eligible women having infants aged less than two years through a multi-stage random sampling method. Personal interview, using structured questionnaire, to collect information on socio-demographic characteristics, antenatal care services, women’s lactation practices, complementary feeding practices and knowledge about BF.

**Results:**

All the included mothers had breastfed their infants, and 32.4% of them initiated BF within the first hour of life and 29.9% exclusively breastfed their infants for 6 months after birth. Complementary feeding was introduced for children aged 6-9 months in 63.6% of them. Bivariate analysis showed that factors favoring exclusive BF were age of the mother (<25 years), with secondary or higher education, number of children, with no history of complicated pregnancy or lactation problems, received health education about BF and having knowledge about BF. Logistic regression model showed that most influential significant predictor for exclusive BF was receiving of health education about BF and adequate knowledge of BF.

**Conclusions:**

Although all rural Egyptian mothers included, initiated BF, the rate of its exclusivity was low. Comprehensive education about BF during pregnancy is strongly needed to promote BF among them.

## 1. Introduction

Breastfeeding (BF) is well recognized as the most appropriate mode for infant feeding. Providing several short- and long-term benefits for both infants and mothers, it is closely related to optimal physical and psychomotor development and chronic disease prevention (WHO, 2009; Jones et al., 2003). For several reasons, BF epidemiology is crucial for an understanding of the health outcomes shaped by the nutrition transition currently underway in many developing countries (WHO, 2010). Virtually all mothers can breastfeed, provided they have accurate information, the support of their family, the health care system and the society at large (WHO, 2009). Exclusive breastfeeding (EBF) is recommended up to 6 months of age, with continued BF along with appropriate complementary food up to two years of age or beyond. BF is one of the most effective ways to ensure child health and survival. Optimal BF together with complementary feeding prevent malnutrition and can save about a million child lives (Jones et al., 2003). According to the W.H.O recommendations, BF should begin within an hour of birth and should be “on demand” while bottles or pacifiers should be avoided (WHO, 2010). In Egypt many efforts have been directed to promote child’s health; one of them was, “Healthy Mother, Healthy Child HMHC (1993-2009)” project which undertook the task of reducing the risk factors for maternal and neonatal mortality and significantly improved outcomes in nine governorates of Upper Egypt; a region that traditionally has been associated with the worst health statistics (Egypt Healthy Mother/Healthy child, 2009).

This package of services combined the best practices with the promotion of behaviors and interventions that are essential for saving lives and reducing morbidity rates among women and children. One important integral of the mentioned project was the Integrated Management of Childhood Illness (UNICEF, 2012) and its primary component included improving child health through promoting exclusive BF for 6 months, promoting healthy diet for BF mothers, providing postnatal supplements and promoting accessibility to supplements for vulnerable mothers at low prices (El-Zanaty & Ann, 2009). Despite the implementation of several programs aiming to promote BF among Egyptian mothers, very few studies (Hossain et al., 1994; Mohamed & Ahmed, 2011) have tackled the determinants of BF exclusivity among mothers in rural Egyptian communities, especially following the epidemiological transition witnessed by the country and the Middle Eastern region. This cross-sectional study aimed to assess BF indicators specifically exclusivity and the timely complementary feeding, while assessing potential determinants that affect exclusivity of BF among a sample of mothers inhabiting Egyptian rural communities. The results of this study could be of potential benefit to support BF programs directed towards this population in the future.

## 2. Methods

### 2.1 Settings and Design

This community based cross-sectional study was conducted over a period of 4 months from February to May 2012 including women having infants aged less than two years targeting mothers at rural Egyptian communities.

### 2.2 Sample Size and Sampling Methods

Considering a prevalence of exclusive BF at the age of 6 months of 30% (El-Zanaty & Ann, 2009) with a power of 80% and alpha level of 5%, the minimum sample size required would be 632, one third of the minimal sample size was added to compensate for possible non-response, the final sample size was amounted to 822 mothers. A multi-stage random sampling technique was employed to select the participants. The first stage involved random selection of two governorates from Lower Egypt namely Giza (about 120 Km from Cairo) and El Fayom (about 190 Km from Cairo). Sampling frame consisting of all villages in the two governorates was prepared and from each, one village was chosen. Both villages have similar socio-demographic profiles and they are served by rural health care units (N=5) delivering basic primary health care services and populated with 21324 (10206 women at the reproductive age) and 8400 (4016 women) (CAPMAS, 2013). Both villages were composed of several satellites (aggregate of rural houses called Ezbas, usually comprised 20 to 50 houses). A second frame was prepared to randomly select twelve Ezbas, five from Giza village and seven from Fayoum village, all eligible women (having children aged two years or less) were indentified through the local health visitors; one thousand women had agreed to participate.

### 2.3 Data Collection

Selected mothers were approached personally through a household survey interviewed by well trained interviewers, with semi-structured pre-tested questionnaire, the items of which were adopted from the available literature (El-Zanaty & Ann, 2009; Hossain et al., 1994; Mohamed & Ahmed, 2011) and designed to collect information regarding:

- Socioeconomic and demographic characteristics of the women and their partners,
- Obstetric and lactation history: history of pregnancy-related problems, pattern of receiving antenatal care (ANC), place of delivery and lactation problems.
- Knowledge of BF and complementary feeding practices were also included: five closed ended items were used to elicit their knowledge about meaning or definition of optimum time to initiate BF, importance of BF to the mother and the baby, exclusive BF, recommended duration of EBF, complementary feeding practices including age at introduction.

### 2.4 Definitions

The used BF indicators, in reference to the World Health Organization recommendations (WHO, 2010):

Timely first-suckling rate: the proportion of infants <12 months of age who first suckled within 1 hour of birth.
Ever-breastfed rate: Is the proportion of infants < 12 months of age who were ever breastfed.
Exclusive breastfeeding rate: That is, the infant had received only breast milk from his/her mother, no other liquids or solids with the exception of drops or syrups consisting of vitamins, mineral supplements or medicines for the first six month of life.
Continued breastfeeding rate (2 years):Is the proportion of children 20–23 months of age who were breastfed.
Timely complementary feeding rate: Is the proportion of infants 6–9 months of age who were receiving breast milk and complementary food.

### 2.5 Data Processing and Analysis

Data were analyzed using Statistical Package for Social Science software (SPSS version 15.0 Inc., IL. USA). Descriptive and inferential statistics were used to summarize and compare the data as appropriate. The Odds ratio (OR) and 95% confidence intervals (CI) was used to assess the strength of association between independent socio-demographic and health-related factors and the prevalence of EBF and also the complementary feeding practices of the study sample. Predictor variables with p value of < 0.05 in the bivariate analysis were included in the logistic regression model. Logistic regression model was performed to detect independent predictors significant at the univariate level for exclusive BF (the dependent variable).

Knowledge scoring was assessed and summed; correct answers scored one point for each question with a maximum score of five points. P-value of < 0.05 was considered as significant.

### 2.6 Ethical Approval

Study protocol was reviewed and approved by the ethical committee at the Community Medicine Department, Faculty of Medicine, Cairo University. Permission to conduct the study in the local area was obtained from the District Medical Officers and informal leaders at the selected rural areas. Eligible Women received proper orientation about the study and its aim with an emphasis of their right of not to participate, those agreed to participate were given written consent forms.

## 3. Results

Table 1 depicts the distribution of the study sample according to their socio-demographic characteristics. The mean age ± standard deviation [SD] of the included mothers was 25±4.6 years. About 43% of them were within the age category of 15≤25 years. More than half (56%) were illiterates; three quarters (76.2%) were housewives. The majority of them (96.1%) were married and 74.1% had more than one child. Almost half of their partner’s (59.9%) of them had secondary/higher education. Fifty one percent of the respondents’ children were males. Forty-five of the studied group conducted 2-3 antenatal care visits. Normal vaginal deliveries were the mode of child birth in about three quarters of them. All mothers included had ever breastfed their baby, one third of the women (32.4%) reported breastfeeding their infants within the first hour after delivery. Ninety six percent of the included mothers had exclusively breastfed their infants during the first month of age, the corresponding figures for the 2^nd^, 3^rd^, 4^th^ and 5^th^ were 83%, 69%, 50% and 39% respectively. And 29.9% had exclusively breastfed their infants for 6 months after birth. Complementary feeding for children aged from 6-9 months was given by 63.7% of the respondents mothers. Only 16.1% of them continued BF their infants for 20-24 months after birth.

**Table 1:** Socio-demographic characteristics of the included mothers at rural Egyptian communities

Characteristic	Frequency (N=1000)	Percentage
**Mother’s age**		
15-<25 years	438	43.8
25-<35 years	394	39.4
35 or more	168	16.8
**Mother’s education**		
Illiterate/read-write	560	56.0
Primary education	215	21.5
Secondary/higher education	225	22.5
**Mother’s employment**		
Housewives	762	76.2
Employed *	238	23.8
**Marital status**		
Married	961	96.1
Widowed/divorced	39	3.9
Parity: Median (mean/SD)	Median 2	Mean 2.23±0.95
**Number of living child**		
Median (mean/SD)	Median 2	Mean1.9± 0.7
One	259	25.9
2 children	632	63.1
3-5	103	10.3
>5 children	6	0.6
**Father’s education**		
Illiterate/read-write	269	26.9
Primary education	132	13.2
Secondary/higher education	599	59.9
**Number of antenatal care visits**		
None	16	1.6
Once	28	2.8
2-3 times	453	45.3
>4 times	503	50.3
**Mode of delivery**		
Normal Vaginal	723	72.3
Cesarean section	277	27.7
Pregnancy-related problems **		
Yes *	223	22.3
No	777	77.7
**History of contraception**		
None	218	21.8
Oral	301	30.1
Intrauterine device	481	48.1
**Gender of the neonate**		
Male	510	51.0
Female	490	49.0
**Health education towards breast feeding**		
None	661	66.1
Once	167	16.7
Twice or more	172	17.2
**Lactational problems**		
None **	569	56.9
Engorgement	296	29.6
Abscess	129	12.9
Others	6	0.6

* Pregnancy related problems included: hypertensive disorders, anemia, prematurity.

** Lactation-related problems included: breast engorgement, breast abscess, sore nipples.

Table 2 displays the distribution of the infants up to 6 months of age (n=187) and shows the correlation between EBF and socio demographic characteristics and health care related factors of the participants. Women in the age group (15-≤25) years constituted 73.2% of the EBF group and this was significantly higher than those in the age group (>25) years (26.8%) (P=0.01). Also women having secondary/higher education were more likely to exclusively breastfed their infants and constituted almost 60% of that group compared to those having primary education or illiterates (39.3%, P<0.01). Mothers having one child were more likely to EBF (67.9%) compared to those with more than one child 32.1% (P=0.03). In addition, women with history of complicated pregnancy of their current child had a significantly lower rate of EBF compared to those who did not constituting 11.6% of EBF group (P=0.04). Among those with lactation problems only seventeen women (30.4%) exclusively breastfed their infants and this rate were significantly lower than without (69.6%) (P=0.02). Women with knowledge score ≥2 (out of 5 points) constituted only 23.2% of the EBF group and this was statistically different from those who scored less than two points (P<0.03)

**Table 2:** Univariate analysis of correlates for breastfeeding exclusivity among the included rural mothers

Characteristic	Exclusive Breast feeding: No. (%)		Univariate analysis	

	Yes (N=56)	None: (N= 131)	Odds Ratio (95% Confidence Intervals)	P value
**Mother’s age**				0.01
15- <25 years	41(73.2)	116(88.5)	Reference	
≥ 25	15(26.8)	15(11.5)	2.8 (1.2-6.2)	
**Mother’s education**				0.0001
Secondary/higher education	22(39.3)	41(31.3)	Reference	
Less	34(60.7)	90(68.7)	3.4 (1.7-6.5)	
**Mother’s employment**				0.31
Housewives	43 (76.8)	91(69.5)	1.4 (0.7- 2.9)	
Employed	13(23.2)	40(30.5)	Reference	
**Number of living child**				0.03
One child	38(67.9)	107(81.7)	0.4 (0.2-1.0)	
Two or more	18(32.1)	24(18.3)	Reference	
**Number of antenatal care**				0.90
One or more	36 (64.3)	85(64.9)	1.0(0.5- 1.8)	
None	20 (35.7)	46(35.1)	Reference	
**Pregnancy-related problems**				0.04
Yes	11(19.6)	45(34.4)	0.4(0.2-0.9)	
**History of contraception**				0.10
Yes	45 (80.4)	90(68.7)	1.8 (0.8- 3.9)	
No	11(19.6)	41(31.3)	Reference	
**Mode of delivery**				0.80
Normal Vaginal	37(66.1)	85(64.9)	Reference	
Cesarean section	19(33.9)	46(35.1)	1.05 (0.5- 2.03)	
**Gender of the neonate**				0.30
Male	32(57.1)	64(48.9)	1.4 (0.7 – 2.6)	
Female	24(42.1)	67(51.1)	Reference	
**Health educationbreast feeding**				0.0001
Yes	38(67.9)	25(19.1)	8.9 (4.4 -18.2)	
No	18(32.1)	106 (80.9)	Reference	
**Knowledge score**				0.030
≥ 2 points	13(23.2)	14(10.7)	2.5 (1.1- 5.8)	
<2 points	43(76.2)	117 (89.3)	Reference	
**Lactationalproblems**				0.020
None	39(69.6)	67(51.1)	Reference	
Yes	17(30.4)	64(48.9)	2.2 (1.1-4.3)	

Table 3 demonstrates the responses of the included mothers about the knowledge items and distributed in relation to educational status and exclusivity of BF. Mothers with higher educational status had significant more correct responses along the domains of benefits of BF, duration of EBF, and complementary feeding, while early initiation of BF and optimum duration of EBF were significantly higher among women practiced EBF.

**Table 3:** Correct responses of the included mothers about breastfeeding knowledge items distributed by educational status and exclusivity of breast feeding

Knowledge items	Educational status: No. (%)			Exclusivity of breastfeeding: no. (%)		

	Secondary or higher (N= 449)	<Secondary (N=551)	P value	Yes	No	P value
1- Benefits of breast feeding **(at least one)**	313 (71.1)	334 (59.7)	0.00	30 (53.6)	57 (43.5)	0.20
2- Initiation of breast feeding: option **(immediately after birth)**	43 (9.8)	118 (21.1)	0.01	40 (71.4)	74 (56.5)	0.05
3- Definition of exclusive breastfeeding	144 (32.7)	243 (43.5)	0.01	25 (44.6)	67 (51.1)	0.4
4- Duration of exclusive breastfeeding	123 (28.0)	111 (19.9)	0.03	14 (25.0)	52 (39.7)	0.05
5- Correct time at introduction of complementary feeding	151 (34.3)	49 (26.7)	0.01	29 (51.8)	75 (57.8)	0.5
**Total knowledge score (out of five)** Median Mean (SD)						
Median	2	2		2	2	
Mean (SD)	2.4± 1.0	2.5 ± 0.9	0.50	2.4 ± 1.2	2.5 ± 0.8	0.90

Regarding the timely feeding practices Table 4 shows that, out of the total mothers with children aged 6 to 9 months (n=196), 125 had effective complementary feeding practice. Correlating both socio-demographic, health related factors and level of awareness with effective complementary feeding practice shows that only the absence of lactational problems was statistically different between mothers who practiced timely complementary breast feeding and those who did not (p-value= 0.03).

**Table 4:** Introduction of complementary feeding distributed by the included socio-demographic and health-related factors among the included mothers

Variables	Complementary feeding (No=196)		Univariate analysis	

	Yes (N=125)	None: (N=71)	Odds Ratio (95% Confidence Intervals)	P value
**Mother’s age**				0.80
15=<25 years	23 (18.4)	14(19.7)	0.9 (0.4-1.9)	
>25	102(81.6)	57(80.3)	Reference	
**Mother’s education**				0.07
Secondary/higher education	91(72.8)	20(28.6)	6.7(3.5-12.9)	
Less	34(27.2)	50 (71.4)	Reference	
**Mother’s employment**				0.10
Housewives	100(80.0)	63(88.7)	0.5 (0.2-1.2)	
Employed *	25(20.0)	8(11.3)	Reference	
**Number of living child**				0.70
One child	90(72.0)	53(74.6)	0.8 (0.45-1.7)	
More	35(28.0)	18(25.4)	Reference	
**Number of antenatal care**				0.40
One or more	52(41.6)	34(47.9)	0.7 (0.4-1.4)	
Non	73(58.4)	37(52.1)	Reference	
Pregnancy-related problems **				0.90
Yes	16(12.8)	9(12.7)	1.01 (0.4- 2.4	
**Mode of delivery**				0.40
Normal Vaginal	107(85.6)	64(90.1)	0.6(0.3- 1.6)	
Cesarean section	18(14.4)	7(9.9)	Reference	
**Health education about breastfeeding**				1.0
Yes	86(68.8)	41(57.7)	1.6 (0.9-3.0)	
No	39(31.2)	30(42.3)	Reference	
**Knowledge score**				0.40
More than or equal 2	71 (56.8)	36 (50.7)	1.3 (0.7-2.3)	
<2	46 (36.8)	35(49.3)	Reference	
**Lactationalproblems**				0.03
No	79(56.8)	55(77.5)	0.5 (0.3-1.0)	
Yes	46(36.8)	16(22.5)	Reference	

Table 5 displays the logistic regression model for exclusive BF. Variables that were significant positive predictors for EBF at the bivariate analysis (p *<* 0.05) were considered as independent predictors in the logistic regression model for EBF. The regression retained seven predictors of EBF including; women aged 15-<25 years, with secondary/higher education, having one child, with no history of complicated pregnancy, no lactation problems, received health education and more knowledgeable regarding BF. Out of these factors, maternal age< 25 years having one child, receiving health education about BF and more knowledgeable about BF were the significant positive predictors to EBF.

**Table 5:** Multivariate regression analysis models for breast feeding exclusivity and timely introduction of complementary feeding

Independent variables	Exclusive breast feeding	

	Odds ratio (95% confidence intervals)	P value
Maternal age **(<25 years)**	3.4 (1.3- 9.3)	0.02
Education **(secondary or higher)**	1.5 (0.5- 3.8)	0.44
Number of children **(one)**	0.3 (0.14-0.9)	0.02
Pregnancy related problems **(none)**	1.5 (0.5-4.3)	0.40
Lactational problems **(none)**	0.8 (0.3-2.1)	0.60
Health education about breast feeding **(yes)**	9.4 (4.0-22.4)	0.001
Knowledge score **(≥2 points)**	2.2 (1.3-3.6)	0.005
Model statistics:		
Percent predicted	79.1%	
P value	0.9	
Chi-square	3.4	

## 4. Discussion

This study revealed that BF was initiated among all rural Egyptian women, nevertheless, the rate of exclusivity was still lower than recommended by the W.H.O. Several predictors were interplayed in shaping the pattern of EBF among them including, the educational status, the health services provided, and adequate knowledge about BF. Egypt is one of the developing countries in which we need to support correct feeding practices. Breastfeeding is a safe, economical, and emotionally satisfying means of feeding babies. The infant feeding indicators functioned in this research were based on standard definitions formulated by a WHO (2009) expert panel. In the current study 32.2% of women initiated breastfeeding in the first hour of labor; these results are higher than those found by El-Gilany, Sarraf and Al-Wehady (2012) whose corresponding results were 11.4%, but lower than those results found among Quatrain women 57% of whom reported early initiation of BF. No agreement between the different studies could be due to different socio-demographic profiles between countries. The current study showed that the proportion of children who were exclusively breastfed for 6 months was 29.9%, which was less than the national average figure of 38% and still well below the 90% level recommended by the WHO (WHO, 2009; El Zanaty & Ann, 2009; Mohamed & Ahmed, 2011). It was however, higher than the EBF prevalence reported from Zimbabwe (7%), Bauru, Brazil (24.2%) and Nigeria (27%) respectively (Parizoto, Parada, Venancio, & Carvalhaes, 2009; Carvalhaes, Parada, & Costa, 2007; Salami, 2006). These results showed wide variation of EBF prevalence between and within countries and over time. Different methodologies for estimating the rate of EBF may also influence the results (Carvalhaes et al., 2007; Desmond, 2008; Salami, 2006). Several factors were found to interplay in the exclusivity of breastfeeding. Our study delineated that some sociodemographic factors-maternal age and education, health-related factors—pregnancy and lactational problems, the level of awareness and health education about breastfeeding and higher knowledge scores may influence exclusivity of breastfeeding. Women belonging to the age group 15-≤25 showed a significantly higher percentage of EBF compared to older group 73.2 versus 26.8%. This could be explained by the fact that the former has eagerness to perform the act of motherhood. This was consistent with Nkala and Msuya (2011) who reported that women 15-24 years old EBF their infants at a higher percentage 61.5% versus 54.1%. Several studies from developing countries have shown that within countries, BF is more prolonged among non-educated women rather than women higher levels of formal education (Trussel et al., 1992; Pe’rez, 2003). These results are in agreement with ours and could be explained by the fact that countries with a higher proportion of their population living in rural areas with lower education rates especially among girls present more extended BF compared with more urbanized nations (Pe’rez, 2003; Monteiro, Conde, & Popkin, 2001). Health education (HE) during pregnancy was an important factor that may explain the finding of significant higher rates of EBF among women who were exposed to HE during their ANC visits versus those who did not. In the present study, adequate knowledge of EBF significantly affected the prevalence of EBF; the higher the level of adequate knowledge of BF among women, the higher the prevalence of EBF. Wen, Baur, Rissel, Alperstein and Simpson in 2009 advocated educating mothers to increase their breastfeeding background influencing their EBF practice. Studying the knowledge assessment parameters, the current study found that illiterate mothers and those with education lesser than secondary, lacked appropriate knowledge compared to women having secondary or higher education in more than half of the studied parameters for assessing knowledge. Abdul Ameer, Al-Hadi and Abdulla (2008) found that illiterate mothers and those with informal or unknown education lacked appropriate knowledge compared to educated women in almost all parameters studied except for frequency of BF. In the same concept Amin, Halbas and AbdAl-Qader (2011) recommended raising the knowledge of Arabian women through proposed policies to promote BF which will expand the awareness of the benefits of breastfeeding to include a larger sample of the community through social clubs and the curricula of schools. Maternal age younger than 25, health education and adequate maternal knowledge about BFwere positive predictors about EBF domain after applying multiple logistic regression model. While Nkala and Msuya in 2011 reported that adequate knowledge, absence of pregnancy problems and delivery at a healthcare facility to be of statistical significant affection upon EBF practices. Similarly Al-Binali in his Saudi Arabian study conducted in 2012 pointed out to the role of knowledge about BF upon BF practices.

## 5. Conclusion

Although all mothers initiated breastfeeding, the rate of exclusive breastfeeding was low at six months. Comprehensive education about breastfeeding problems and its’ management, as well as education on positioning and attachment, should be offered to women during pregnancy and immediately after delivery in this setting. This will build up a background which will help in facing any problem they may encounter in the future during breastfeeding of their kids, not simply to stop breastfeeding or introduce wrong complementary foods prematurely.

Nation-wide qualitative studies are required in this setting to explore the prevalence of EBF as well as complementary feeding practices reported, suggesting the factors hindering optimum breastfeeding practices and recommending approaches which could manage these factors.
